# Regulation of Lignin Biosynthesis by Post-translational Protein Modifications

**DOI:** 10.3389/fpls.2020.00914

**Published:** 2020-07-02

**Authors:** Daniel B. Sulis, Jack P. Wang

**Affiliations:** Forest Biotechnology Group, Department of Forestry and Environmental Resources, North Carolina State University, Raleigh, NC, United States

**Keywords:** lignin, lignification, proteins, PTMs, SCW, trees, wood formation

## Abstract

Post-translational modification of proteins exerts essential roles in many biological processes in plants. The function of these chemical modifications has been extensively characterized in many physiological processes, but how these modifications regulate lignin biosynthesis for wood formation remained largely unknown. Over the past decade, post-translational modification of several proteins has been associated with lignification. Phosphorylation, ubiquitination, glycosylation, and S-nitrosylation of transcription factors, monolignol enzymes, and peroxidases were shown to have primordial roles in the regulation of lignin biosynthesis. The main discoveries of post-translational modifications in lignin biosynthesis are discussed in this review.

## Introduction

Wood is a biological composite and a valuable source of feedstock for construction timber, pulp and paper, and biofuels (Wang et al., 2018). Wood is composed of secondary xylem tissue formed by secondary cell walls (SCW) (Fromm, 2013). The secondary xylem consists of two types of cells: fibers, which provide mechanical support, and tracheary elements composed of vessels (not found in gymnosperm wood) and tracheids (found in both angiosperm and gymnosperm) for water and solutes transport (Demura and Fukuda, 2007). SCW is mainly composed of cellulose, hemicelluloses, and lignin, in different ratios of these constituents (Li et al., 2011; Ye and Zhong, 2015).

Lignin is a phenolic polymer formed by phenylpropanoid monomeric units, 4-coumaryl alcohol (H-unit), coniferyl alcohol (G-unit), and sinapyl alcohol (S-unit), also known as monolignols. The biosynthesis of lignin occurs in several consecutive reactions involving up to 11 different enzyme families and 24 metabolites (Wang et al., 2019b). The pathway is complex and regulated by a network of substrates and inhibitors in the conversion of phenylalanine or tyrosine to monolignols (Wang et al., 2014). The monolignols are then transported to the lignifying zone and oxidized by peroxidases and laccases to phenoxy radicals (Li et al., 2014). Many efforts have been made to elucidate the regulation of the lignin biosynthetic pathway. Transcription factors (TFs) associated with lignification have been identified for *Pinus* (Patzlaff et al., 2003a, b), *Eucalyptus* (Goicoechea et al., 2005; Legay et al., 2010), and *Populus* (Karpinska et al., 2004; Zhong et al., 2010b; Ohtani et al., 2011; Li et al., 2012, 2015; Tian et al., 2013; Wang S. et al., 2015; Yang et al., 2017; Chen et al., 2019; Gui et al., 2019; Zheng et al., 2019) tree species. These TFs impart transactivation and transrepression of monolignol genes and other TFs in complex hierarchical transcriptional regulatory networks. Some TFs associated with monolignol biosynthesis form protein-protein (TF-TF) interactions that may affect TF-target DNA binding (Chen et al., 2019). Lignification is also regulated at the enzyme level. Monolignol biosynthetic enzymes can directly interact with each other (e.g., Ptr4CL3-Ptr4CL5, PtrC3H-PtrC4H, At4CL1-AtC3H, At4CL1-AtC4H, and AtCCR1-AtC4H interactions) (Chen et al., 2011; Chen H. -C. et al., 2014; Gou et al., 2018; Wang et al., 2019a) or indirectly through common mediators such as AtMSPBs (e.g., AtC3H-AtMSPBs, AtC4H-AtMSPBs, AtF5H-AtMSPBs interactions) (Gou et al., 2018). In addition, monolignol biosynthetic enzymes can interact with proteins in other biological pathways (e.g., OsCCR1-OsRac1, ZmCCoAOMT/ZmHCT-ZmRp1 interactions) (Kawasaki et al., 2006; Wang G. H. et al., 2015; Wang and Balint-Kurti, 2016). These interactions may modulate enzyme stability, activity, and metabolic flux in lignin biosynthesis, increase the biosynthesis of lignin in response to pathogen infection, and suppress plant hypersensitive response (Kawasaki et al., 2006; Chen et al., 2011; Chen H. -C. et al., 2014; Wang G. H. et al., 2015; Wang et al., 2019a; Wang and Balint-Kurti, 2016; Gou et al., 2018). However, the role of post-translational modifications (PTMs) in SCW formation remains poorly understood.

PTMs are covalent processes that alter the properties of proteins by proteolytic cleavage or addition of modifying groups to one or more amino acids (Mann and Jensen, 2003). Over 200 different types of PTMs have been identified, ranging from small chemical modifications (e.g., phosphorylation and acetylation) to the addition of complete proteins (e.g., ubiquitination) (Beltrao et al., 2013; Spoel, 2018). PTMs are essential for many proteins and can affect the localization, stability, structure, activity, and molecular interactions of proteins (Nørregaard Jensen, 2004; Wang J. P. et al., 2015). In plants, PTMs are associated with plant growth and development, biotic and abiotic stress response, and metabolism (Catala et al., 2007; Mazzucotelli et al., 2008; Stulemeijer and Joosten, 2008; Miura and Hasegawa, 2010; Friso and van Wijk, 2015). Phosphorylation of cellulose synthases and cellulose synthase-like proteins were identified to have essential roles in regulating the activity and distribution of cellulose synthase complexes (CSCs) along microtubules (Speicher et al., 2018). The ubiquity of PTMs and their diverse regulatory functions is indicative of the complexity of secondary cell wall biosynthesis in general, and lignin biosynthesis in particular (Table 1).

**TABLE 1:** PTMs of proteins involved in lignification.

Protein	Organism	Types of PTM	Detection of PTM	Effect of PTM	References
PAL2	*P. vulgaris* (French bean)	Phosphorylation	*In vitro*	Unknown	Allwood et al., 1999
PAL2	*P. trichocarpa x P. deltoides*	Phosphorylation	*In vitro*	Phosphorylation of PAL2 decreases Km and V_max_	Allwood et al., 1999; Cheng et al., 2001
AldOMT2	*P. trichocarpa*	Phosphorylation	*In vitro*	Phosphorylations of AldOMT2 reduce enzyme activity	Wang J. P. et al., 2015
MYB4	*P .taeda*	Phosphorylation	*In vitro*	Phosphorylation of MYB4 reduces the MYB4-transactivation activity over the lignin target genes	Morse et al., 2009
RAI1	*O. sativa*	Phosphorylation	*In vitro*	Phosphorylation activates RAI1 and increases the expression of PAL1.	Kawasaki et al., 2006; Kim et al., 2012; Akamatsu et al., 2013; Nasir et al., 2018
LTF1	*P. trichocarpa*	Phosphorylation	*In vivo* and *In vitro*	Phosphorylation reduces the LTF1 stability via 26S proteasome and reduces the LTF1-transrepression activity over the lignin target genes	Gui et al., 2019
PAL1-4	*A. thaliana*	Ubiquitination	*In vivo*	Ubiquitination reduces the protein stability via 26S proteasome	Zhang et al., 2013
CCR	*O. sativa*	Ubiquitination	Untested	Interaction of OsCCR with SCF^OsFBK1^ reduces the OsCCR stability via 26S proteasome	Borah and Khurana, 2018
MYB156	*P. tomentosa*	Ubiquitination	Untested	Interaction of MYB156 with UBC34 reduces the MYB156-transactivation over the lignin genes possibly decreasing the MYB156 stability via 26S proteasome	Zheng et al., 2019
MYB221	*P. tomentosa*	Ubiquitination	Untested	Interaction of MYB221 with UBC34 reduces the MYB221-transactivation over the lignin genes possibly decreasing the MYB221 stability via 26S proteasome	Zheng et al., 2019
VND7	*A. thaliana*	S-nitrosylation	*In vitro*	S-nitrosylation of VND7 decreases the VND7-transactvation activity over the SCW genes	Kawabe et al., 2018
VND7	*A. thaliana*	Ubiquitination	Untested	VND7 protein accumulates upon treatment with MG−132 in transformed tobacco BY−2 cells	Yamaguchi et al., 2008
PXP1-6	*P. trichocarpa*	Glycosylation	*In vivo*	Unknown	Christensen et al., 1998
PRX	*Z. elegans*	Glycosylation	*In vitro*	Glycosylations change the PRX catalytic efficiencies.	Gabaldón et al., 2005, 2006, 2007

## Phosphorylation of Monolignol Biosynthetic Enzymes

Lignin biosynthesis is typically perceived as a matrix of linear or branched enzymatic reactions that successively modify the aromatic ring of the phenylpropane units and conversion of the side-chain carboxyl to an alcohol moiety. The involvement of many enzymes suggests that the pathway is well coordinated to mediate precise control of the rate and ratios of monolignol biosynthesis for polymerization. PTMs provide an efficient way to impart spatiotemporal activation and inactivation of monolignol enzyme activities in plants. However, the identification of PTMs involved in lignin biosynthesis remains challenging. Some studies based on phosphoproteomics could not reliably detect phosphopeptides for monolignol enzymes in wood forming cells (Mauriat et al., 2015). The difficulty of detecting protein phosphorylation is in part due to the highly dynamic nature of the regulatory mechanism.

## PAL Phosphorylation

Phenylalanine ammonia-lyase (PAL) is a family of enzymes that catalyzes the deamination of phenylalanine to cinnamic acid, representing the first reaction step in the phenylpropanoid pathway (Shi et al., 2013; Zhang and Liu, 2015). PAL enzymes have been extensively characterized for their protein structure, functionality, and primordial role in wood formation (Ritter and Schulz, 2004; Shi et al., 2013). Some evidence regarding the chemical modification of PAL has been proposed. In *Phaseolus vulgaris* (French Bean) suspension-cultured cells, a phosphopeptide derived from PAL2 was detected (Allwood et al., 1999). The authors suggested that the kinase responsible for the PAL2 phosphorylation belongs to the calmodulin-like domain protein kinase (CDPK) family (Allwood et al., 1999). Consistently, a recombinant PAL2 protein from *P. trichocarpa* × *P. deltoides* was phosphorylated by CDPK derived from French bean suspensions and Arabidopsis (Allwood et al., 1999; Cheng et al., 2001). In PAL of *Phyllostachys edulis*, *in silico* functional analysis indicated a likely presence of nine casein kinase phosphorylation sites, eight protein kinase C phosphorylation sites, and two N-glycosylation sites (Gao et al., 2012). The high degree of PAL peptide sequence similarity across plant species and their conserved phosphorylation by CDPK may suggest a common phosphoregulatory mechanism for PAL in phenylpropanoid biosynthesis (Allwood et al., 1999; Cheng et al., 2001; Zhang and Liu, 2015).

The functional role of post-translational phosphorylation in PAL remains to be clarified. Allwood et al. (1999) showed that one hour after co-incubation of PAL2 and CDPK, the Km and V_max_ of PAL2 is slightly reduced. On the other hand, longer co-incubation of up to 4 h reduced the protein stability of the phosphorylated isoform of PAL2 and result in a 3-fold reduction in the V_max_ when compared to the non-phosphorylated isoform (Allwood et al., 1999). The phosphorylation of PAL2 likely limits the rate of phenylalanine conversion to cinnamic acid, thereby influencing the metabolic flux for lignification.

**FIGURE 1:**
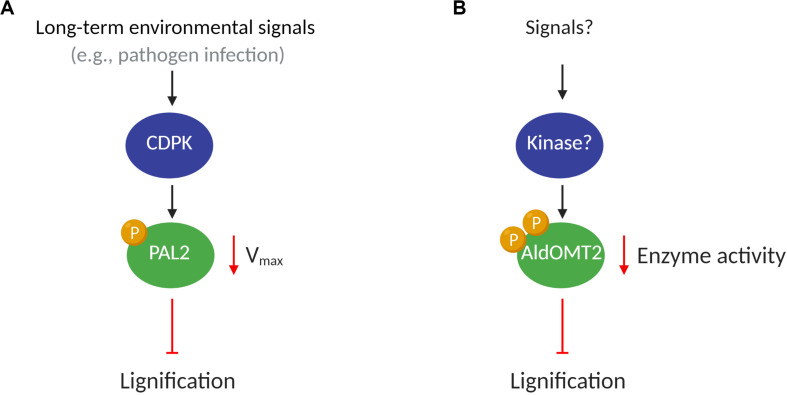
A putative model of kinases regulating monolignol enzymes activity. **(A)** Under potential stimulus such as pathogen infections, the CDPK proteins phosphorylate PAL2, decreasing the V_max_ of the enzyme and reducing lignification in plants; **(B)** Kinase(s) protein(s), after an endogenous or environmental stimulus, phosphorylates AldOMT2 at either of the two phosphorylation sites and negatively regulates the AldOMT2 activity.

The CDPK family of kinases has been associated with the regulation of biological processes encompassing plant growth, development, and response to biotic and abiotic stresses (Romeis, 2001; Schulz et al., 2013). Pathogen infections induce the expression and activity of PAL (Jones, 1984; Shoresh and Harman, 2008), which in turn promotes immune response mediated by salicylic acid production, enhanced physical barriers (lignin), and accumulation of antimicrobial compounds (e.g., phytoalexins) (Jones, 1984; Solecka, 1997; Chen et al., 2009; Adams-Phillips et al., 2010; Hamann, 2012; Yan and Dong, 2014). Since PAL transcript expression is upregulated under biotic stress, the CDPK-mediated phosphorylation of PAL may mark it for turnover, thus reducing PAL activity (Allwood et al., 1999) to maintain homeostasis (Figure 1A). The phosphorylation of PAL has also been suggested to translocate the proteins to membranes, which may contribute to metabolic channeling in lignin biosynthesis (Allwood et al., 1999; Rasmussen and Dixon, 1999).

## AldOMT2 Phosphorylation

5-Hydroxyconiferaldehyde *O*-methyltransferase 2 (AldOMT2) catalyzes the 3/5-methylation of caffeoyl- and 5-hydroxyferuloyl–containing acids, aldehydes, and alcohols for monolignol biosynthesis. In *P. trichocarpa*. *PtrAldOMT2* is the highest transcribed gene in the monolignol biosynthetic pathway and the third-highest transcribed gene in stem differentiating xylem (SDX). The protein abundance of PtrAldOMT2 is also the highest of all monolignol enzymes, accounting for 5.9% of the SDX proteome (Shuford et al., 2012; Lin et al., 2013). Given the abundance in transcripts and proteins of PtrAldOMT2, regulation of its activity by transcriptional control is energy-intensive. In contrast, regulation of PtrAldOMT2 activity by protein phosphorylation removes the need to synthesize/degrade RNA and protein, thus providing a rapid and energetically efficient mode of regulation for O-methyltransferase activity (Wang J. P. et al., 2015). Phosphoproteomic analysis by mass-spectrometry in SDX revealed two phosphopeptides in PtrAldOMT2 that contain either a phosphoserine at peptide position-123 or position-125. Concurrent phosphorylation of both Serine123 and Serine125 was not detected *in vivo*. The phosphorylation of PtrAldOMT2 is mediated by kinases in the SDX protein extract and could be reversed by phosphatase treatment (Wang J. P. et al., 2015). Phosphorylation reduced the enzymatic activity of recombinant PtrAldOMT2 considerably, and this reduction in activity was not due to protein degradation (Wang J. P. et al., 2015). Moreover, site-directed mutagenesis by replacing either Serine123 or Serine125 with asparagine, a negatively charged amino acid that mimics the properties of phosphoserine, showed that Serine123 is more critical for phosphoregulation of enzyme activity. Serine123 is conserved in 43 of 46 (93%) AldOMTs across diverse plant species (Wang J. P. et al., 2015), suggesting a conserved evolutionary phosphoregulation of the enzyme. The presence of two phosphoserine residues in PtrAldOMT2 and their distinct enzymatic regulations provide a basis for further exploring mechanistic insights to phosphoregulation in monolignol biosynthesis. Whether different kinases and signaling pathways regulate the two phosphorylation sites or if the sites are functionally redundant, remains to be determined (Wang J. P. et al., 2015). Lignification is regulated by many environmental and developmental stimuli that combinatorially modulate the rate and ratios of monolignol biosynthesis. The understanding of how PTMs transduce the regulation to changes in metabolic flux for monolignol biosynthesis would provide valuable knowledge of plant adaptation and cell wall biosynthesis (Figure 1B).

## Phosphorylation of TFs in Lignin Biosynthesis

R2R3-MYBs are TFs that contain a DNA binding domain at the N-terminus, which is composed of two imperfect helix-turn-helix repeats of approximately 50 amino acids (R2 and R3) (Jin and Martin, 1999; Patzlaff et al., 2003a). Protein members from the R2R3-MYB family of TFs are known to bind at AC rich cis-elements present in the promoters of many genes in the lignin biosynthetic pathway (Lois et al., 1989; Joos and Hahlbrock, 1992; Leyva et al., 1992; Hauffe et al., 1993; Hatton et al., 1995; Logemann et al., 1995; Bell-Lelong et al., 1997; Séguin et al., 1997; Lacombe et al., 2000; Lauvergeat et al., 2002; Patzlaff et al., 2003a). Some R2R3-MYBs have been identified and characterized as key regulators of plant cell wall biosynthesis (Patzlaff et al., 2003a). PtMYB4 is an ortholog of AtMYB56 and AtMYB83 (Patzlaff et al., 2003a; Yang et al., 2016; Gui et al., 2019), which function as second layer transactivators of SCW biosynthesis in Arabidopsis (Yang et al., 2016). The overexpression of *PtMYB4* in tobacco induced monolignol biosynthesis and promoted lignin accumulation in non-lignified cell types, suggesting that this TF regulates positively the lignin biosynthesis genes during the wood formation process (Patzlaff et al., 2003a).

Using a cDNA library from *Pinus taeda* SDX, Morse et al. (2009) screened MAPKs (PtMAPK6 and PtMAPK13) potentially involved in wood formation. *In vitro* phosphorylation assays demonstrated that PtMAPK6 could phosphorylate PtMYB1 and PtMYB4, transregulators of lignin biosynthesis in SDX. Replacement of Lysine68 for arginine abolished the kinase activity of PtMAPK6, demonstrating that the lysine is essential for mediating target phosphorylation. Serine236 was identified to be the phosphorylation site in PtMYB4. Replacement of Serine236 for a glutamate residue (mimicking constitutive phosphorylation of PtMYB4) did not interfere with the affinity for DNA binding but significantly reduced the transactivation of the target gene (Morse et al., 2009).

**FIGURE 2:**
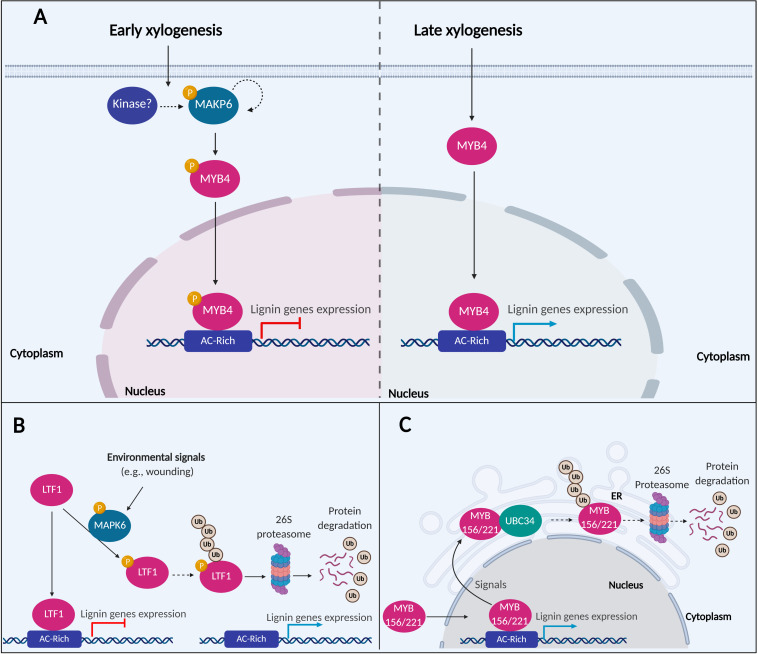
A putative model of MAPK6 regulating the activation of lignin biosynthesis. **(A)** In the early xylogenesis stage in *P. taeda*, MAPK6 proteins are autophosphorylated or activated by other kinase-mediated phosphorylation. MAPK6 becomes activated and phosphorylates the TF MYB4. Phosphorylation inactivates MYB4, resulting in the repression of lignin genes. In the late xylogenesis stage, MAPK6 is no longer active, and MYB4 induces the expression of lignin genes **(B)** In *P. trichocarpa*, LTF1 is a repressor of lignin genes under normal conditions. After environmental stimuli such as wounding, MAPK6 can interact and phosphorylate LTF1. Phosphorylation destabilizes LTF1 in the cells and promotes its degradation via 26S proteasome and attenuating the repression of lignin genes mediated by LRF1. **(C)** A putative model of MYB156 and MYB221 regulating the activation of lignin biosynthesis in *P. tomentosa*. MYB156 and MYB221 are repressors TFs of lignin genes. UBC34 ubiquitin-conjugating enzyme interacts with MYB156 and MYB221 and alters the subcellular localization of the TFs from the nucleus to the ER. The expression of the lignin genes are attenuated either by the TFs trapping into ER or translocation of the MYB156 and MYB221 either traps or degradation via ubiquitin and 26S proteasome pathway. The dotted arrows indicated hypothesized events not confirmed experimentally.

MAPKs are components of signal transduction networks that trigger a variety of biological processes in plants (Nühse et al., 2000). MAPK6 is associated with response to pathogen infection, ethylene response, and cell wall biosynthesis and remodeling (Andreasson and Ellis, 2010; Seifert and Blaukopf, 2010; Bacete et al., 2018). All these processes affect secondary cell wall biosynthesis for wood formation (Adams-Phillips et al., 2010; Fromm, 2013; Li et al., 2015; Ye and Zhong, 2015). In *Oriza sativa*, OsMAPK3 and OsMAPK6 are involved in the phosphorylation of OsRAI1 through a complex signaling cascade upon chitin elicitation. The activation of OsRAI1 by phosphorylation induces the transcript expression of defense-associated genes including *PAL1* (Kawasaki et al., 2006; Kim et al., 2012; Akamatsu et al., 2013; Nasir et al., 2018). In *P. taeda*, PtMAPK6 is expressed in cell differentiation stages 1, 2 (division and expansion), and 3, 4 (SCW synthesis) of SDX, but its kinase activity for PtMYB4 phosphorylation was mainly observed in the initial stages of 1 and 2. The phosphorylated isoform of PtMAPK6, representing the active form of the enzyme, was only found in stages 1 and 2 (Morse et al., 2009). PtMAPK6 may be autophosphorylated or activated by other kinase-mediated phosphorylation during early xylogenesis. The activated PtMAPK6 then phosphorylates PtMYB4, reducing the TF transregulation of cell wall biosynthetic genes (Figure 2A). PtMAPK6 activity during early xylogenesis may function to suppress the expression of PtMYB4-regulated genes from resulting in premature deposition of lignin in dividing and expanding cells. The absence of PtMAPK6 activity in the late stages of differentiating xylem allows PtMYB4 to transactivate lignin biosynthetic genes in these cells (Morse et al., 2009).

LTF1 is the closest ortholog of AtMYB4 in *P. deltoides* × *P. euramericana* (Gui et al., 2019). Arabidopsis *AtMYB4* mutants showed increased expression of *C4H* and reduced expression of *CCoAOMT*. Overexpression of *AtMYB4* in tobacco increased the expression of *CCoAOMT* and reduced the expression of *C4H, 4CL*, and *CAD* genes (Jin et al., 2000). In *P. deltoides* × *P. euramericana*, LTF1 is mainly expressed in the SDX as a transrepressor of monolignol biosynthetic genes, including *4CL*. *LTF1* mutants in poplar showed elevated transcript expression of lignin genes and increased lignin content. The overexpression of *LTF1* in transgenic poplar showed opposite effects, decreasing the expression of lignin genes and reducing the overall lignin content. *LTF1* overexpression induced dwarfism in 2-month old poplar (grown in phytotron), but the severity of dwarfism was reduced when the plants reached eight months old (Gui et al., 2019). The protein abundance of LTF1 was decreased significantly in 8-month-old plants. Phosphoproteomic analysis identified two phosphopeptides corresponding to LTF1 in 8-month old plants. These phosphopeptides were not detected in the 2-month-old *LTF1*-overexpressed plants. Disruption of the phosphorylation sites in transgenic plants exhibited reduced plant height, stem diameter, and internode length, and reduced lignin deposition in the fiber cells of these plants, and xylem vessel collapse (Gui et al., 2019). Recombinant LTF1 proteins were rapidly degraded when co-incubated with SDX extracts, and the addition of proteasome inhibitor MG–132 re-establishes the initial levels LTF1. On the other hand, even in the absence of MG–132, the levels of LTF1 protein containing mutations in the phosphorylation sites remained stable after the incubation with the SDX extracts.

PdMAPK6 in *P. deltoides* × *P. euramericana* is a homolog of the Arabidopsis and *P. taeda* MAPK6, identified by protein-protein interactions to be associated with LTF1 (Morse et al., 2009; Gui et al., 2019). *In vitro* phosphorylation assays showed that PdMAPK6 could phosphorylate LTF1 at the Threonine146 and Threonine178 positions, while mutagenesis of these phosphorylation sites prevented LTF1 phosphorylation. A higher level of phosphorylated LTF1 and other PdMAPK6 activated proteins were detected in 8-month old greenhouse-grown poplar, compared to 2-month old plants grown in indoor growth chambers. Greenhouse-grown plants are more susceptible to environmental signals like biotic and abiotic stress (Gui et al., 2019), consistent with the functions of MAPK6 in pathogenic response (Andreasson and Ellis, 2010; Bacete et al., 2018). In this context, after mechanical wounding, increased expression of lignin genes and improved lignin deposition were detected in plants overexpressing *LTF1*. However, these effects were significantly lower in plants overexpressing *LTF1* without the phosphorylation sites. Consistently, the expression of Arabidopsis ortholog *AtMYB4* is repressed by environmental stimuli, including UV-B and wounding (Jin et al., 2000). These data suggest that LTF1 is a repressor of lignin genes, reducing the lignin content in plant cells. Under environmental stimuli, LTF1 is phosphorylated, decreasing its stability by degradation via 26S proteasome (Gui et al., 2019; Figure 2B).

The high peptide sequence identity of PdMAKP6 and PtMAKP6 (84%) and the similar phosphoregulation of MYB TFs suggest an important role of MAPK in the regulation of lignin biosynthesis in both angiosperm and gymnosperm species. Future studies focusing on MYB phosphorylation by MAPK6 and their interaction with external stimuli would better inform the phosphoregulation of lignin biosynthesis. For instance, is mechanical wounding the only stimulus of LTF1 phosphorylation, or are other signals such as ethylene response, cell wall biosynthesis, and cell remodeling also involved? PTMs are found in diverse classes of proteins, highlighting the regulatory interplay among PTMs as a common strategy to regulate protein functions (Yang, 2005; Nussinov et al., 2012; Lothrop et al., 2013; Pejaver et al., 2014). Other types of PTMs could also be involved in the regulation of MYB TFs. Identifying the upstream regulators of MAPK6 and other wood TFs phosphorylated by MAPK6 are needed to determine the network of regulatory interactions by kinases in the lignification of tree species.

## Ubiquitination of PAL

Ubiquitination is a common regulatory mechanism in all eukaryotes that target proteins for degradation via the 26S proteasome, thereby maintaining protein turnover in the cells. The ubiquitin attachment process involves three enzymes: the E1 ubiquitin-activating enzyme, the E2 ubiquitin-conjugating enzyme, and an E3 ubiquitin ligase (Guo and Yang, 2017). Proteins containing a well-conserved 40–50 amino acid F-box domain in the N-terminal region are referred to as F-box proteins that are one part of the ubiquitin E3 ligase complex. F-box proteins are involved in many physiological processes in plants, such as flowering, pathogen defenses, circadian cycle, and phytohormones signaling (Kuroda et al., 2002). PAL enzymes from Arabidopsis (PAL1, PAL2, PAL3, and PAL4) have been shown to interact with Kelch domain-containing F-box (KFB) proteins (KFB01, KFB20, KFB39, and KFB50) (Zhang et al., 2013; Zhang et al., 2015). The F-box domain in KFBs that confers ubiquitin ligase activity is associated with reducing PAL protein abundance by decreasing protein stability (Zhang et al., 2013). Co-overexpression of *PALs*-*GFP* and *KFBs* in Arabidopsis reduced the conversion efficiency of phenylalanine to t-cinnamic acid by up to 80% (Zhang et al., 2013). More recently, a protein called Small and Glossy Leaves 1 (SAGL1) was identified to be involved in the PTM regulation PAL1. Phylogenetic analysis using 99 Arabidopsis KFB homologous proteins revealed that SAGL1 is closely related to the PAL-regulating KFBs (KFB01, KFB20, KFB39, and KFB50), but located in a separate clade from these KFBs (Yu et al., 2019). SAGL1 can interact and reduce the stability of PAL1, leading to reduced PAL activity for monolignol biosynthesis. Similar to other KFB-mediated PALs regulation, the level of PAL1 was fully restored after MG–132 treatment for proteasome inhibition (Yu et al., 2019).

KFB and SAGL1 have similar regulatory roles in PAL protein stability. Incubation of recombinant PAL in protein extracts of 6-week old Arabidopsis stems led to a significant reduction in the abundance of PAL proteins. The addition of the MG–132 proteasome inhibitor to the assay could maintain PAL protein abundance by preventing ubiquitination-based protein degradation (Zhang et al., 2013). Double and triple mutants in Arabidopsis for the *KFB01*, *KFB20*, and *KFB50* genes showed an increased amount of PAL proteins, and consequently, more acetyl-bromide lignin in the plant cell walls. Overexpression of *KFBs* genes, in contrast, caused a 2 to 70% lignin reduction in the transgenics (Zhang et al., 2013). *SAGL1* mutants in Arabidopsis showed a strong purple color in rosette leaves, leaf petioles, and inflorescence stems, typically found where high amounts of anthocyanin are accumulated (Nakatsuka and Nishihara, 2010; Yu et al., 2019). Quantification of lignin content in the mature stems of the mutants showed a 2-fold increase in lignin content compared to wild-type. The *SAGL1* mutants also had a 60% increased conversion rate of L-phenylalanine to trans-cinnamate, compared to wild-type. PAL enzyme activity increased in *SAGL1* mutants without changing the transcript levels of the *PAL* genes (Yu et al., 2019). Transgenic lines overexpressing *SAGL1* showed significant reductions in the conversion rate of L-phenylalanine to trans-cinnamate and lignin content in the mature stems compared to the wild-type (Yu et al., 2019).

The reduced abundance of PAL proteins in Arabidopsis overexpressing *KFBs* or *SAGL1* was not due to reduced transcript levels of *PAL* genes. In contrast, increased transcript abundance of *PALs* were observed in the transgenic lines overexpressing *KFBs*, possibly to compensate for the rapid turnover of PAL proteins in the cells (Zhang et al., 2013). The discrepancy between PAL transcript and protein abundances in the transgenics is indicative of cross-talks between transcriptional regulation and PTM to maintain homeostasis for lignin biosynthesis. PAL mediates the entry step in the phenylpropanoid pathway and is extensively regulated for metabolic control (Zhang and Liu, 2015). In *O. sativa*, an auxin-responsive Kelch-domain-containing F-box protein (OsFBK1) interacts with cinnamoyl-CoA reductase (OsCCR) to mediate its protein degradation via the proteasome pathway, thereby regulating lignin content in the cell walls (Borah and Khurana, 2018). This suggests that the ubiquitin E3 ligase complex proteins may interact with other lignin biosynthetic enzymes. In *P. trichocarpa*, 337 F-box proteins were identified using a bioinformatics approach (Schumann et al., 2011), suggesting a possible role of these proteins in the regulation of turnover rate in monolignol enzymes. The involvement of this class of protein in regulating lignin biosynthesis and wood formation in tree species remains to be validated.

## S-Nitrosylation and Ubiquitination of TFs

TFs containing the NAM/ATAF/CUC (NAC) domain are key regulators of plant development, senescence, SCW formation, and biotic and abiotic stress responses (Olsen et al., 2005; Nakashima et al., 2007; Puranik et al., 2012; Chen X. et al., 2014). Several members of the NAC family of TFs have been identified and characterized in Arabidopsis, rice, soybean, wheat, poplar, and citrus (Puranik et al., 2012). In *O. sativa*, pathogen infection induces the phosphorylation of OsNAC4, leading to its accumulation in the cell nucleus. OsNAC4 is involved in the transregulation of 139 genes and hypersensitive cell death (Kaneda et al., 2009), but its association with SWC biosynthesis has not been reported. Seven *NAC* genes are highly transcribed during vessel cell differentiation in Arabidopsis (Kubo, 2005). *VND7* has been extensively characterized as a transregulator of protoxylem vessel development (Kubo, 2005; Yamaguchi et al., 2010). Repression of *VND7* and *VND6* using plant repression domain (SRDX) resulted in defects in Arabidopsis growth and vessel formation, mediated by repressed central metaxylem vessel formation and central protoxylem vessel formation (Kubo, 2005). Induced expression of *VND7* accelerated vessel cell differentiation in all cell types encompassing cotyledon, leaf, hypocotyl, and root cells (Yamaguchi et al., 2010; Kawabe et al., 2018).

The knockout of S-nitrosoglutathione reductase 1 (*GSNOR1*) in transgenic Arabidopsis expressing *VND7* resulted in mutant seedlings lacking xylem vessel differentiation (mutant “suppressor of ectopic vessel cell differentiation induced by VND7” or *seiv*) (Yamaguchi et al., 2010; Kawabe et al., 2018). Indeed, the inducible-*VND7* expression in the *seiv* mutants did not show any evidence of cell wall deposition. These mutant seedlings showed a dwarf phenotype, suggesting that the *GSNOR1* gene may be involved in plant growth and development, in addition to regulating VND7 for xylem trans-differentiation (Kawabe et al., 2018). VND7 directly or indirectly regulates the expression of genes involved in SCW formation during xylem vessel differentiation, encompassing the biosynthesis of cellulose, hemicellulose, and lignin, and programed cell death (Yamaguchi et al., 2011). The expression of these VND7-downstream genes is induced by *VND7* overexpression, and suppressed in *seiv* mutants, demonstrating that the knockout of *GSNOR1* disrupts VND7-mediated regulation (Kawabe et al., 2018). Biotin switch assays showed that the VND7 was S-nitrosylated *in vitro*, and amino acid substitution of two Cysteine264 and Cysteine320 abolished the S-nitrosylation signals. The presence of S-nitrosoglutathione reduced VND7 activity in Arabidopsis protoplasts. VND7 activity was reduced when either Cysteine264 or Cysteine320 were substituted for tryptophan, which mimics a constitutive S-nitrosylated isoform of VND7. Thus, S-nitrosylation plays an essential role in negatively regulating VND7 activity for vessel development. However, replacing the Cysteine264 or Cysteine320 for a serine residue, which mimics a non-S-nitrosylated form of VND7, also caused a reduction in the transactivation activity of VND7 (Kawabe et al., 2018). Cysteine residues in plant proteins are modified by different types of PTMs in addition to S-nitrosylation, including S-sulfenylation, S-glutathionylation, sulfinylation, sulfonylation, and the formation of intra-/intermolecular disulfide bridges (Waszczak et al., 2015; Kawabe et al., 2018). Therefore, other PTMs may be regulating VND7 and causing the non-S-nitrosylated isoform of VND7 to reduce transactivation activity in protoplasts (Kawabe et al., 2018).

NAC-domain proteins have been demonstrated to interact with many cellular components involved in different cellular processes (Yamaguchi et al., 2008), making them a potential target for protein modifications. For instance, the RING domain protein SINA of Arabidopsis 5 (SINAT5) interacts with NAC1 and promotes its degradation via the ubiquitin/26S proteasome pathway (Olsen et al., 2005). Similarly, VND7 is regulated by 26S proteasome-mediated degradation (Yamaguchi et al., 2008). Protein extracts from transformed tobacco BY-2 cells expressing *VND7* had low levels of detected VND7 protein after 24 h, while the addition of proteasome inhibitor increased the abundance of VND7 (Yamaguchi et al., 2008).

Although herbaceous plants such as Arabidopsis have a limited secondary growth compared to tree species, some similarities may be found in the general organization of their tissues (Chaffey et al., 2002; Demura and Fukuda, 2007; Zhong et al., 2010b). Repeated removal of inflorescent stems in Arabidopsis can induce some secondary xylem production that has been used in developmental studies of secondary growth (Oh et al., 2003). Searching for NAC TFs in the *P. trichocarpa* genome, Zhong et al. (2010b) identified 16 homologous genes of Arabidopsis *VND*, *NST*, and *SMB* involved in secondary wall biosynthesis or xylem differentiation. These poplar genes were named *PtrWNDs* for “wood-associated NAC domain transcription factors” (Zhong et al., 2010b; Ohtani et al., 2011). Of the 16 genes, 12 showed a high level of transcript expression in vessel and fiber cells of developing woody tissue. Moreover, they were localized in the nucleus of plant cells and exhibited transactivation activities, consistent with their putative function as TFs (Zhong et al., 2010b). Expression of *PtrWNDs* in Arabidopsis *snd1 (nst3) nst1* double mutants rescued the pendent inflorescence stem phenotype and restored stem strength and deposition of secondary walls in interfascicular fibers. Overexpression of *PtrWND2B* and *PtrWND6B* (sub members of the *SMB* and *VND* class of TF, respectively) in Arabidopsis increased the transcript expression of cellulose synthases (*CesA4*, *CesA7*, and *CesA8*), xylan biosynthetic genes (*FRA8*, *IRX8*, and *IRX9*), and lignin biosynthetic genes (*4CL1* and *CCoAOMT1*) (Zhong et al., 2010b; Ohtani et al., 2011). PtrWND2B and PtrWND6B also transactivated wood-associated TFs involved in the biosynthesis of lignin, hemicelluloses, and cellulose (Zhong et al., 2010b). Overexpression of 12 *PtVNS*/*PtrWND* in *P. trichocarpa* caused SCW formation surrounding the transgenics’ vascular tissue, similarly to which was seen for the induced expression of *VND7* in Arabidopsis and poplar epidermal cells (Ohtani et al., 2011). Expression of *VND7* in poplar induced the expression of many genes related to SCW formation, including enzyme-encoding genes and TFs (Ohtani et al., 2011). Taken together, NAC TFs in Arabidopsis and *P. trichocarpa* are functionally important for SCW formation (Zhong et al., 2010a; Ohtani et al., 2011). Orthologous proteins are typically conserved in their PTMs, and the level of conservation is higher in closely related species (Remmerie et al., 2011; Venne et al., 2014). PTMs identified for Arabidopsis NAC TFs, such as S-nitrosylation and ubiquitination, may also occur in PtrWNDs in the regulation of wood formation.

In *P. tomentosa*, PtoMYB156 and PtoMYB221 from the subgroup 4 of the R2R3-MYB TF family work as transrepressors of lignin genes (Zheng et al., 2019). Overexpression of *PtoMYB156* dramatically retarded plant growth and reduced secondary xylem formation, while thickening of SCWs in the vascular stem tissue was observed in *PtoMYB156* knockout plants (Yang et al., 2017). The regulation of PtoMYB156 and PtoMYB221 is mediated by interactions with an E2 ubiquitin-conjugating enzyme 34 (PtoUBC34), which translocate the TFs to the ER (Zheng et al., 2019). In mesophyll protoplasts of *P. tomentosa* overexpressing PtoMYB156 and PtoMYB221, the TFs are localized in the cell nucleus and could suppress the transcript expression of target genes in the absence of PtoUBC34. When PtoUBC34 is co-expressed in the same system, PtoMYB156 and PtoMYB221 are translocated to the ER in a dose-depend way, and their repression activity is reduced (Zheng et al., 2019). Whether the reduced repression activity of PtoMYB156 and PtoMYB221 is due to the trapping of these TFs in the ER or degradation via the 26S proteasome pathway remains unknown (Figure 2C). Lignin biosynthesis responds to various developmental and environmental signals such as light, sugar content, circadian rhythms, plant hormones, and wounding, where these signals are converted to a physiological process by hierarchical transcriptional regulation of lignin pathway genes (Zheng et al., 2019). The translocation to cellular compartments or possibly ubiquitination and degradation via the 26S proteasome pathway may be a pivotal point in regulating the activity of these TFs. Future studies will help to clarify the cross-talking of lignin biosynthesis and other physiological processes (Zheng et al., 2019).

## Glycosylation in Peroxidases

Class III peroxidases are present as large multigenic families in all land plants (Mathé et al., 2010). Generally, this class of proteins is heme-containing enzymes, which may oxidize a wide range of substrates using hydrogen peroxide as a reduction agent. Peroxidases are associated with multiple cellular processes, encompassing primary and secondary metabolism, hormone catabolism, pathogen defense, phenol oxidation, cross-linking of cell wall-structural proteins, and polysaccharides, and lignin polymerization (Christensen et al., 1998). As aforementioned, peroxidases oxidize the monolignols in the plant cell walls to free radicals for conjugating to the growing lignin polymer (Li et al., 2014). The oxidation reaction mediated by lignin peroxidases is characterized by a three-step cycle involving a two-electron enzyme oxidation: FeIII + H_2_O_2_ → CoI + H_2_O (*k1*); followed by two one-electron reductions: (i) CoI + RH → CoII + R^⋅^; (*k2*) and (ii) CoII + RH → FeIII + R^⋅^; (*k3*), where FeIII, CoI, CoII, and RH are the native enzyme, compound I, compound II, and the monolignol, respectively (Gabaldón et al., 2006).

Most of the peroxidases are glycosylated, and the carbohydrate content may constitute up to 25% of the protein (Christensen et al., 1998; Veitch, 2004) Glycosylation sites are normally found in the sequence motif asn-x-ser/thr (where x means any amino acid residue), and they occur within surface turns or loop regions connecting helices (Veitch, 2004). Six anionic stem peroxidases (PXP1-6) were isolated from xylem tissues of *P. trichocarpa*, and all were heavily glycosylated, exhibiting N-glucosamine and ten putative glycosylation sites in their structures (Christensen et al., 1998). Similarly, two isoforms of ZePrx, where one was fully glycosylated (ZePrx 34.70) and another partially glycosylated (ZePrx 33.44) were identified in *Zinnia elegans* suspensions cells (Gabaldón et al., 2005; Gabaldón et al., 2007). The two isoforms of this protein showed different biochemical properties. For instance, the *k1* (CoI formation constant – which monitors the reactivity of the enzyme with H_2_O_2_) was higher in the ZePrx 34.70 compared to ZePrx 33.44 in the presence of either *p*-coumaryl alcohol or coniferyl alcohol substrates. On the other hand, the opposite effects were observed when coniferaldehyde, sinapyl alcohol, and sinapaldehyde were used as substrates. Moreover, the *k3*, (CoII reduction constant – which monitors the ability of the oxidized form of the enzyme, CoII, to oxidize phenolics) was higher for ZePrx 33.44 comparing to the ZePrx 34.70 in the presence of coniferyl alcohol. Again, the opposite effects were observed in the presence of sinapyl alcohol, where the reactivity was higher for the ZePrx 34.70 (Gabaldón et al., 2006). The different reactivity rates suggest that glycans modulate the catalytic activity of the peroxidases (Gabaldón et al., 2006). Since multiple glycosylation sites are found in these enzymes, the different glycosylation patterns may determine the substrate specificity for monolignol oxidation, leading to changes in the composition of the lignin. Glycosylation may have a role beyond the regulation of peroxidase substrate specificity. Protein folding and stabilization, and protein-cell wall interactions have also been predicted as putative functions of this PTM in peroxidases (Schuller et al., 1996; Gabaldón et al., 2007; Cesarino et al., 2012).

## Final Considerations

Despite the technical challenges in the detection and characterization of PTMs *in vivo*, recent discoveries have provided insights into their importance in regulating lignin biosynthesis and wood formation. The lignin biosynthetic pathway is complex and controlled by many external and developmental stimuli. The complexity of the regulations suggests that TFs, monolignol enzymes, and peroxidases could respond rapidly and specifically to different types of stimuli in the modulation of lignin biosynthesis. Phosphorylation (e.g., PAL2 and AldOMT2) and ubiquitination (e.g., PALs and CCR) of monolignol enzymes decrease the enzymatic activity and protein stability (Allwood et al., 1999; Zhang et al., 2013; Wang J. P. et al., 2015; Borah and Khurana, 2018). More comprehensive identification of PTMs associated with lignin biosynthesis will further improve our understanding of the spatiotemporal regulation of the pathway, and how such regulations coordinately affect the properties of the cell walls. MAPKs mediated MYB4 and LTF1 phosphorylation (Morse et al., 2009; Gui et al., 2019) have provided valuable mechanistic insights on how PTM modulates the SCW but the complete signaling pathway and the interactions between MAPKs and other MAPK-cascade components remain to be elucidated. Further studies focusing on how developmental and environmental signals trigger the MAPK-cascade to activate or repress TFs regulating lignin biosynthesis would be valuable to determine the network of regulatory interactions that control phenotypic alterations.

Protein degradation via 26S proteasome (e.g., VND7, LTF1, and possibly MYB156 and MYB221) plays an essential role in regulating TFs for lignin biosynthesis (Kawabe et al., 2018; Gui et al., 2019; Zheng et al., 2019). However, there are knowledge gaps to be filled for a better understand of how TF protein turnover is modulated by PTMs. Ubiquitination, which is related to the 26S proteasome pathway, remains to be identified in LTF1, MYB156, and MYB221. Moreover, the combinatorial effects of multiple PTMs and their coordinated control of lignin biosynthesis remain poorly understood. For example, the glycosylation of lignin peroxidases (e.g., ZePrx) controls the enzyme substrate specificity (Gabaldón et al., 2006), and further studies may elucidate how multiple glycosylations, and glycosylation together with other PTMs may combinatorially affect lignin composition and structure. Moreover, the relationship between VND7 S-nitrosylation and other types of cysteine modifications in the transregulation of SCW genes is unknown (Kawabe et al., 2018). One PTM can influence the modification of other types of PTMs, which can result in a broad variation of possible proteoforms (Friso and van Wijk, 2015). The combinatorial effect of multiple PTMs and cross-talk between PTMs in the same protein, or on different proteins within complexes, is crucial to defining the interrelationships of multiple PTMs in lignin biosynthesis.

Much of the early work on plant PTMs have focused on model organisms such as Arabidopsis. These PTM in woody species, particularly in the regulation of lignin biosynthesis has remained largely unknown. Due to the large variation in lignin content, composition, and structure between Arabidopsis and perennial woody species, the extent of similarity or difference in the SCW-associated PTMs across species should be evaluated. Systematic co-expression analysis of SCW protein abundances with PTM enzymes during SDX formation, or genome-scale protein interactomics could reveal putative PTM regulatory interactions controlling lignin biosynthesis. The resulting interactions may guide future studies to verify whether these PTMs occur *in vitro* and *in vivo* and how they regulate lignin biosynthesis and wood formation. Comprehensive identification of PTMs in trees will be crucial to understanding the transduction of complex regulatory information that mediates wood formation.

## Author Contributions

DS and JW conceptualized and literary reviewed and wrote the manuscript.

## Conflict of Interest

The authors declare that the research was conducted in the absence of any commercial or financial relationships that could be construed as a potential conflict of interest.
